# Eye Tracking the Feedback Assigned to Undergraduate Students in a Digital Assessment Game

**DOI:** 10.3389/fpsyg.2019.01931

**Published:** 2019-09-06

**Authors:** Maria Cutumisu, Krystle-Lee Turgeon, Tasbire Saiyera, Steven Chuong, Lydia Marion González Esparza, Rob MacDonald, Vasyl Kokhan

**Affiliations:** Department of Educational Psychology, Centre for Research in Applied Measurement and Evaluation, University of Alberta, Edmonton, AB, Canada

**Keywords:** eye tracking, eye movement, error processing, feedback, game-based assessment

## Abstract

High-quality feedback exerts a crucial influence on learning new skills and it is one of the most common psychological interventions. However, knowing how to deliver feedback effectively is challenging for educators in both traditional and online classroom environments. This study uses psychophysiological methodology to investigate attention allocation to different feedback valences (i.e., positive and negative feedback), as the eye tracker provides accurate information about individuals’ locus of attention when they process feedback. We collected learning analytics via a behavioral assessment game and eye-movement measures via an eye tracker to infer undergraduate students’ cognitive processing of feedback that is assigned to them after completing a task. The eye movements of *n* = 30 undergraduates at a university in Western Canada were tracked by the EyeLink 1000 Plus eye tracker while they played Posterlet, a digital game-based assessment. In Posterlet, students designed three posters and received critical (negative) or confirmatory (positive) feedback from virtual characters in the game after completing each poster. Analyses showed that, overall, students attended to critical feedback more than to confirmatory feedback, as measured by the time spent on feedback in total, per word, and per letter, and by the number of feedback fixations and revisits. However, there was no difference in dwell time between valences prior to any feedback revisits, suggesting that returning to read critical feedback more often than confirmatory feedback accounts for the overall dwell time difference between valences when feedback is assigned to students. The study summarizes the eye movement record on critical and confirmatory feedback, respectively. Implications of this research include enhancing our understanding of the differential temporal cognitive processing of feedback valences that may ultimately improve the delivery of feedback.

## Introduction

The valence of feedback, positive or negative, has the potential to affect how learners interact with feedback and how they process it. However, it is not clear how learners react when they are assigned a specific feedback valence that they would not necessarily choose. Yet, this is one of the most common scenarios encountered in the classroom: students are often assigned either positive (confirmatory) or negative (critical) feedback with very little choice between these two valences. Although it is suggested in the literature that individuals process disconfirming information more deeply than confirming information (Baumeister et al., 2001), other studies propose that individuals tend to minimize and distance themselves from criticism (Taylor, 1991). Thus, in contrast to behavioral methods, eye-tracking technology may be applied to provide a more precise measurement of feedback processing specific to each feedback valence.

### Confirmatory and Critical Feedback Processing

In education, one of the most frequent psychological interventions is the provision of feedback to the learner (Kluger and DeNisi, 1998), as studies have found a profound impact of feedback on learning performance (Hattie and Timperley, 2007). Delivering effective feedback is an important goal for educators and for advancing feedback theory. However, it is challenging and it requires a deep understanding of the underpinnings of the feedback mechanisms. An extensive meta-analysis found that feedback hindered performance in comparison to no feedback in a third of the educational studies examined (Kluger and DeNisi, 1998). Indeed, although critical (negative) feedback seems more helpful for learning and performance, as it fills a gap between what the individual needs to know and what the individual actually knows, this type of feedback can trigger ego threat or a blow to one’s self-esteem (Kluger and DeNisi, 1998). Concomitantly, confirmatory (positive) feedback can motivate individuals to continue working on a task. For instance, in the context of information integration categorization tasks, it was found that learning declines when feedback is missing from correct trials (Ashby and O’Brien, 2007). However, when it refers to the person (i.e., to self) and not to the task, confirmatory feedback can be more detrimental than no feedback at all.

Research also suggests that criticism is processed for a longer period of time than confirmatory information for a variety of reasons. First, individuals are thought to employ an adjustment process, post-error slowing (PES), which reflects a longer dwell time after an error than after a correct trial (Botvinick et al., 2001). Second, an attentional orienting that detracts an individual’s attention from the task to the error could be attributed to the element of surprise induced by errors (Houtman and Notebaert, 2013), thus leading to PES. Third, in line with the surprise factor, the hypercorrection effect suggests that feedback that is surprising tends to increase an individual’s attention toward that feedback (Butterfield and Metcalfe, 2006). Fourth, self-focused attention adjustments were found to be associated with recruiting more resources to deal with criticism than with confirmatory information when processing social feedback (Buscher et al., 2012; Vanderhasselt et al., 2015). A potential increased processing of critical feedback, compared to confirmatory feedback, may be the first step in elucidating the mechanisms of feedback processing for learning improvement, so further exploration is warranted.

### The Contribution of This Study

This study constitutes a first empirical comparison, using eye movement methodology, between the time spent looking at each feedback valence, critical and confirmatory, when written feedback is assigned to the learner. Previous research has examined the dwell time on critical and confirmatory feedback by assessing students’ actions in the Posterlet game, the assessment instrument employed in the current study. Findings showed that students who had a choice between seeking critical and confirmatory feedback about their posters in Posterlet spent significantly more time dwelling on critical feedback (Cutumisu et al., 2015). However, there are two important distinctions between prior research and the current study. First, in prior research, feedback was chosen by the study participants, rather than being assigned to them as in the present study. Second, in previous research, dwell time was indirectly inferred using behavioral methods via learning analytics from participants’ mouse clicks rather than using physiological methods via an eye tracker to provide more fine-grained temporal information as in the present study. Thus, in prior research it was not possible to determine whether individuals dwelled longer specifically on critical or on confirmatory feedback based solely on the behavioral measures. Consequently, the present study affords the possibility of measuring the precise time spent attending to each feedback valence by employing both behavioral and physiological measures. Also, building on our prior work (Cutumisu et al., 2018), the current study includes more data points and separates the computation of feedback dwell time into the time students took to read the feedback on their first encounter with that feedback (i.e., during the first visit on each feedback box) and the time they took to read the feedback after leaving that feedback box and returning to it (i.e., during subsequent visits on the same feedback box).

This study explores feedback processing when feedback is assigned to learners by examining university students’ eye gazes on critical and confirmatory written feedback while they are playing a poster-design digital game. It hypothesizes that university students dwell longer on critical than on confirmatory feedback when feedback is assigned to them, posing the following research questions:

1. Do participants dwell more on assigned critical feedback than on confirmatory feedback? Do results persist when feedback revisits are discounted?
2. Is the mean number of gaze fixations different between valences of assigned feedback?
3. Is the mean number of feedback revisits different between feedback valences of assigned feedback?

## Literature Review

Eye movement methodology has been used by researchers to scrutinize cognitive processes (Liversedge and Findlay, 2000), including problem solving and reasoning (Poole and Ball, 2006) by tracking in real time participants’ eye gaze locations and their sequence of eye shifting, as eye gaze locations are thought to be associated with the focus of attention. It enables researchers to track two types of eye movements, saccades and fixations. Saccades are continuous, irregular, and rapid gaze moving actions or jumps (e.g., 20 to 50 milliseconds) that individuals take when reading or looking at visual stimuli to obtain as much information as possible about the presented stimuli (Rayner, 1998). Findings suggest that attention can anticipate a saccade on an area of interest and that, in complex tasks (e.g., information processing, such as reading), the target of participants’ attention and their saccades are closely related (Rayner, 1998). Between saccadic movements, the eye will remain relatively stationary and will dwell more on one point in a period of relative immobility (i.e., fixation). Thus, fixations are short gaze stops between saccades and they provide temporal information regarding a person’s gaze (Rayner, 1998). For instance, the duration of a fixation during reading can vary between 100 and 300 milliseconds based on many variables, such as the type of words presented or the complexity level of the text (Rayner, 1998). Fixations measure visual attention and they are used to infer mindful cognitive processing and the integrative, conscious effort of reading feedback (Bolzer et al., 2015). Taken together, these measures provide a window into understanding individuals’ cognitive processes.

Eye movement recordings are employed to provide a dynamic trace of the direction of an individual’s attention in relation to a visual display (Poole and Ball, 2006). Dwell time on areas of interest has been widely used to measure attention, such as in the context of associative learning (Hogarth et al., 2008; Le Pelley et al., 2011; Koenig et al., 2017). Also, in an eye-tracking study that employed a categorization task, although the time spent on the actual feedback was not measured, it was found that participants dwelled longer on the stimulus associated with feedback on incorrect than on correct trials, suggesting the importance of incorrect over correct trials (Watson and Blair, 2008).

Many studies employed eye tracking to investigate cognitive processes, such as reading. Eye tracking can provide information about participants’ allocation of attention, such as whether they are paying attention or whether they are reading the feedback that is provided to them. Eye tracking can become a useful tool when examining written feedback, as it can provide information about how feedback is being processed. Measures such as fixation durations can be used as indicators of attention by providing information regarding the amount of time spent reading feedback. They can also provide useful information to facilitate the comparison of the time spent reading one form of feedback over another. A second useful measure is the number of times participants regress to re-examine feedback (i.e., revisit the feedback). A regression represents an eye movement back to a previously read line or visited object in an area of interest. For example, in a reading task, about 10 to 15% of saccades are regressions on previous words (Rayner, 1998). Different interpretations can be found as to why a participant would regress in a reading task. For example, a student can regress due to misinterpretation or lack of comprehension of the text (Rayner, 1998). If participants decide to return to and read a feedback message again, they might be trying to understand the meaning of the feedback, so that they could integrate it into their current task (e.g., revise their work based on that feedback).

However, there is a paucity of eye tracking literature on feedback processes, especially regarding the impact of online processing of written feedback with the use of eye movement (Timms et al., 2016). The few published studies examined different aspects of feedback, such as how feedback can improve learning outcomes, impact peer-feedback, determine where students allocate their attention and orient their attention toward the goal (Kiili and Ketamo, 2010; Bolzer et al., 2015), and identify when students are bored or disengaged (e.g., in a biology intelligent tutoring system; D’Mello et al., 2012). Other eye-tracker studies investigated if learners look at the feedback offered by online environments and, if so, for how long. In a study sampling Grade 5 and Grade 6 students, researchers measured participants’ eye movement while participants were playing a math computer game. Participants also received hints, so researchers could assess the time students spent on the hints (Conati et al., 2013). This game provided students with different types of hints and feedback (e.g., definitions, tools, and bottom-out hints) throughout the game. Results showed that time spent on hints varied by the type of hint and its presentation frequency. In addition, more time spent carefully reading the hints tended to impact the correctness of participants’ next move. Finally, students who manifested positive affect with respect to help took more time to read the hints. Thus, eye-tracking can provide more detail regarding the way students interact with feedback in an online environment (Conati et al., 2013).

## Materials and Methods

### Participants and Procedure

Participants (*n* = 30, 20 females and 10 males) were undergraduates recruited from a large research-intensive university in Western Canada via a research participation pool program. They ranged from 18 to 32 years of age, with a mean age of M = 22.5 years (SD = 3.92) and M = 16.13 (SD = 2.47) years of education. Ethics approval was secured from the University’s REB board (Pro00059774, “Eye tracking students’ gameplay in Posterlet”) before commencing the study. Students provided written informed consent before participating, received a copy of the completed informed consent form, and also received course credit for their participation. All participants self-identified as having normal or corrected-to-normal vision and not being color blind.

Participants were individually tested in sessions lasting circa 45 min. The first couple of minutes were used to calibrate and then validate the eye tracker using a five-point calibration sequence in which participants had to follow a dot that appeared at five different locations on the computer screen. Calibration is necessary to fine-tune video-based eye trackers, so that the particularities of each participant’s pupil movement can be mapped to screen coordinates. This procedure was repeated until the average deviation of the visual angle was one degree. After that, participants played the Posterlet game for about 15–20 min, followed by an online post-test for up to 20 min.

### Measurement Instruments

Three instruments were employed in this study: the Posterlet digital behavioral assessment game; the EyeLink 1000 Plus eye tracker to capture students’ gazes in the game; and a digital background information post-test.

#### Posterlet: A Computer-Based Behavioral Assessment Game

Posterlet is a computer-based behavioral assessment three-round game in which players design digital posters (Cutumisu et al., 2015). In the game version adapted for this study (i.e., the *Assign* version), participants are assigned feedback on their poster designs. On each level, players design a digital poster by dragging existing text and graphics entities onto the poster’s canvas. Then, players test their poster by receiving feedback from three virtual animal characters. The feedback provided by the game characters is generated by the game’s intelligent feedback system that analyzes the poster against a set of 21 graphic design principles provided by a graphic design artist (Cutumisu et al., 2017). The graphic design principles comprise three categories: essential information (i.e., rules pertaining to the inclusion on the poster canvas of crucial information necessary to attend the fair, such as the date and location of the fair), readability (i.e., rules pertaining to the clarity of the text and images on the poster, such as the text size or image size), and space use (i.e., rules pertaining to the appropriate use of space on the poster canvas, such as the 30–70 principle of proportions). The game alternates between informative (“Your poster helps people know where to go.”) and uninformative (“I really enjoy fairs. I plan on going to this one.”) feedback for the same valence.

**FIGURE 1:**
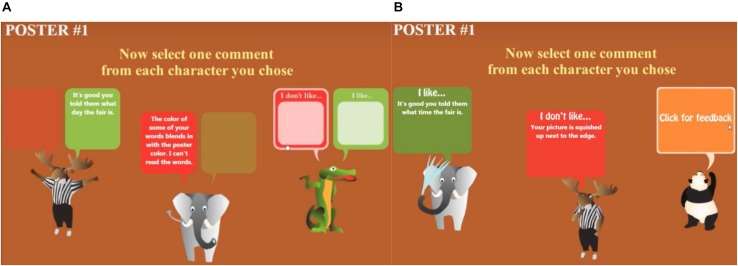
The two Posterlet game versions: the original (Choose) version of the Posterlet game **(A)** and the modified (Assign) version of the Posterlet game **(B)**. The latter game version was employed in the present study.

**FIGURE 2:**
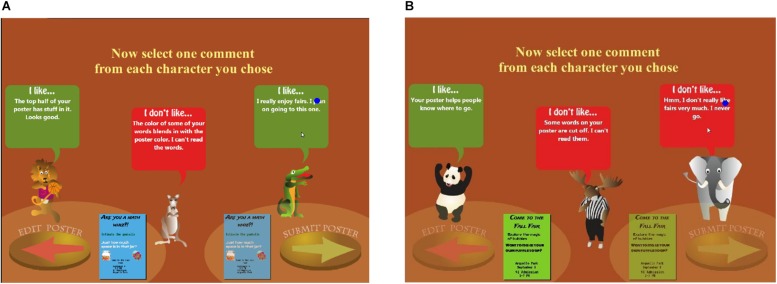
The gaze on the feedback area of a participant in the Assign version of the Posterlet game is represented as a dark-blue dot. The participant reads confirmatory feedback in panel **(A)** (“I really enjoy fairs. I plan on going to this one.”) and critical feedback in panel **(B)** (“Hmm, I don’t really like fairs very much. I never go.”).

The process of selecting feedback in Posterlet is shown in Figure 1. In the original (i.e., Choose) version of the Posterlet game (Figure 2A), after completing the first poster, the participant clicked on a green box (confirmatory feedback from the moose), then on a red box (critical feedback from the elephant) to reveal the feedback, preparing to choose critical feedback from the crocodile. In the modified (i.e., Assign) version of the game, players click on the orange box located above each of the three characters, which they previously chose, to reveal feedback that is either critical or confirmatory, depending on what the matched participant in the Choose condition had previously chosen. This process follows an experimental yoked-study design. In the Assign version of the Posterlet game (Figure 2B), after completing the first poster, the participant clicked on the orange box (“Click for feedback”) above the first two animal characters to reveal the feedback assigned: confirmatory feedback from the elephant and critical feedback from the moose, matching the same feedback valence and order chosen by a participant in the Choose condition illustrated in Figure 2A. The player is preparing to click on the orange box to retrieve the last piece of feedback.

The original Posterlet game was designed to assess players’ willingness to choose negative feedback and to choose to revise their work. Conversely, this study employed an alternative game version (*Assign*) that aimed to mimic a regular situation encountered in schools, where the instructor assigns feedback to students. Thus, in this version of the game, instead of choosing their feedback valence, students were assigned pre-determined feedback valences in a specific order. This allowed the researchers to control the feedback valence assigned to students. Thus, this study constitutes a first step in gaining a deeper understanding of the processes unfolding when students interact with critical feedback, by separating the choice over the feedback valence.

#### Eye Tracker: EyeLink 1000 Plus

The SR Research *EyeLink 1000 Plus* infrared video-based pupil-monitoring eye tracker was employed in this study to record monocular eye movements. The eye tracker was used in a desktop remote mode. The EyeLink 1000 Plus is an eye tracker with a high degree of accuracy (0.5°). It samples gaze locations at a frequency of 500 Hz. This system requires that the camera and an infrared sensor are placed on the desk facing the participant at about 35 cm from the computer screen. Participants placed their foreheads on a headrest to prevent head movements at approximately 81 cm from the computer screen. A target sticker was placed on each participant’s face to track eye movements while compensating for slight head movements. As students played a dynamic computer game in which they were provided with many choices and thus had different experiences, areas of interest could not be defined as in more traditional eye-tracker studies. Hence, their gazes on the game’s areas of interest were recorded using the Screen Recorder software (SR Research, 2019). A student’s gaze is represented as a dark-blue dot in Figure 2. A desktop computer was linked to the experimenter’s computer via an Ethernet cable, allowing real-time feedback regarding participants’ gaze positions and enabling the researcher to detect if the protocol was not followed or if the gaze disappeared (i.e., the participant moved and the eye tracker needed re-calibration). The EyeLink Data Viewer software (SR Research, 2019) was employed for data analyses. This program enables the researcher to see a video of participants’ fixations superimposed on the presented stimuli, as well as quantitative information for each event, including fixation durations and timestamps, mouse movements, and screen coordinates.

#### Post-Test Survey

Participants filled an online post-test immediately after completing the game. The survey included questions about their gender, age, color blindness, and education.

### Measures

This section describes a subset of the behavioral measures collected via the game and online surveys, including the valence of the feedback assigned to students. It also describes a set of physiological measures collected via the eye tracker, including ocular fixation frequency to measure attentional capture as well as fixation duration to measure attentional holding. Step-by-step coding guidelines were created to ensure that data coding was consistent. The eye-tracker data were coded independently by three undergraduate students for each participant, through a time-intensive process lasting over 4 months, based on videos of each session recorded by the Screen Recorder software that complements the Data Viewer software. The Data Viewer could not extract automatically all the information from the live game session (i.e., the same area of interest could not be defined from one participant to the next to extract gaze information).

For each feedback box, students recorded information about the poster as well as about the feedback order, valence, and message. Importantly, students recorded the start timestamps of each event (e.g., mouse up, mouse hover, fixation, etc.) and of each fixation as well as the end time of the last event and of the last fixation, as long as these criteria were met: (1) the student’s gaze was seen on the box (i.e., the dark-blue dot, as that shown in Figure 2, was on the box); (2) the feedback text was revealed in the box; and (3) the gaze did not leave the box. Whenever the gaze left the box, the next time the gaze overlapped with the box, a separate entry was coded, enabling revisits to be accurately tracked. Thus, no recording was made if the student’s gaze was not overlapping with the box or if the student was looking at the box but had not yet clicked to reveal the feedback text (i.e., the “Click for Feedback” text was displayed). The number of fixations and regressions (i.e., revisits) on each feedback message as well as the duration of each fixation were also recorded. Different ways of documenting students’ gazes were employed to corroborate the results. For instance, the time spent on each feedback message was obtained in three ways: (1) the sum of all fixations on that message across all visits; (2) the sum of the differences between the end time of the last fixation and the start time of the first fixation on that message on each visit; (3) the sum of the differences between the end time of the last event and the start time of the first event on that message on each visit.

Then, a doctoral research assistant compared the results for each coded participant and, together with the principal investigator, reconciled all the discrepancies. Later on, three more undergraduate students were asked the code the data. Following that, results were compared with those generated previously and consensus was reached.

#### Feedback

*Critical Feedback* counts the number of negative feedback (“I don’t like”) messages assigned to a participant across the game. *Confirmatory Feedback* counts the number of positive feedback (“I like”) messages assigned to each participant across the game.

These measures range from zero to nine (i.e., three posters × three feedback messages) and are complementary (i.e., they add up to nine).

#### Gazes on Feedback

This measure constitutes the amount of feedback messages where a participant’s gaze was tracked successfully on critical feedback (*Gazes on Critical Feedback*) and confirmatory feedback (*Gazes on Confirmatory Feedback*) across the three posters, respectively. Each of the messages was coded with 1 if a gaze was detected on that feedback message and 0 otherwise. The measure corresponding to each valence ranges from zero to the amount of feedback of that valence assigned in the game.

For example, if a participant is assigned two confirmatory and one critical feedback as illustrated in Figure 2A, it is possible for the gaze on the leftmost confirmatory feedback message to be missing (i.e., the eye tracker failed to track it). Thus, in this scenario, although Confirmatory Feedback is 2 for this poster, Gazes on Feedback is 1. This situation can occur if participants move during the experiment or if they cover the tracking sticker placed on their face, causing the eye tracker to stop tracking their gaze. This measure enables the mean fixation duration per feedback valence to be computed, as the fixation duration for each feedback message is only computed for messages where a participant’s gaze was present.

#### Mean Gaze Duration

This measure constitutes the average time that participants spend looking at each feedback message of critical feedback (*Mean Gaze Duration on Critical Feedback*) or confirmatory feedback (*Mean Gaze Duration on Confirmatory Feedback*), respectively, across the Posterlet game.

#### Mean Gaze Duration per Letter

This measure constitutes the average time that participants spend looking at each letter of feedback per valence (*Mean Gaze Duration per Letter of Critical Feedback* and *Mean Gaze Duration per Letter of Confirmatory Feedback*, respectively) across the Posterlet game. It is an important measure, as it enables a fair comparison of the time taken to attend to each feedback valence, especially because critical and confirmatory feedback may have different lengths. The sum of the individual fixation durations on each feedback message, which included the durations of the regressions (i.e., revisits) on that message, was divided by the length (i.e., the number of letters, including spaces) of the feedback message. Finally, the values of all these measures were added for all the messages of each valence. As a result, two measures were composed across the game: the total time spent on critical feedback per letter and the total time spent on confirmatory feedback per letter. Then, each of these two measures was divided by the *Gazes on Critical Feedback* and by the *Gazes on Confirmatory Feedback*, respectively, to obtain the average time spent per letter of feedback valence.

#### Mean Gaze Duration per Word

This measure constitutes the average time a participant spent looking at each word of critical (*Mean Gaze Duration per Word of Critical Feedback*) and confirmatory (*Mean Gaze Duration per Word of Confirmatory Feedback*) feedback, respectively, across the game.

Similar to the procedure for computing the mean gaze per letter, the total time spent on all feedback messages of each valence was divided by the number of words of each feedback message to obtain an estimate of the average time spent per word of feedback valence.

#### Mean Number of Fixations on Feedback

This measure constitutes the average number of a participant’s gaze fixations on the critical (*Mean Number of Fixations on Critical Feedback*) and confirmatory (*Mean Number of Fixations on Confirmatory Feedback*) feedback messages across the game, respectively.

These measures were also computed by length of feedback (*Mean Number of Fixations on Critical Feedback Per Letter* and *Mean Number of Fixations on Confirmatory Feedback Per Letter*, respectively) and by number of words of feedback (*Mean Number of Fixations on Critical Feedback Per Word* and *Mean Number of Fixations on Confirmatory Feedback Per Word*, respectively).

#### Mean Number of Regressions on Feedback

This measure constitutes the average revisits on critical (*Mean Number of Regressions on Critical Feedback*) and confirmatory (*Mean Number of Regressions on Confirmatory Feedback*) feedback messages, respectively, across the game.

These measures were also computed by length of feedback (*Mean Number of Regressions on Critical Feedback Per Letter* and *Mean Number of Regressions on Confirmatory Feedback Per Letter*, respectively) and by the number of words of feedback (*Mean Number of Regressions on Critical Feedback Per Word* and *Mean Number of Regressions on Confirmatory Feedback Per Word*, respectively).

#### Demographic Information

The information collected from students included gender, age, color blindness, and years in school.

## Results

### Do Participants Dwell More on Assigned Critical Feedback Than on Confirmatory Feedback?

The assembly of the data sources as well as the statistical analyses were carried out using the *R* programming language (R Core Team, 2019). Descriptive analyses were conducted to provide more information about the variables included in this study, as shown in Table 1.

**TABLE 1:** Means and standard deviations of critical and confirmatory feedback assigned per game.

**Measures (*n* = 30)**	**Mean critical (SD)**	**Mean confirmatory (SD)**
Feedback Assigned	5.77 (1.38)	3.23 (1.38)
Gazes on Feedback	5.77 (1.38)	3.23 (1.38)

**FIGURE 3:**
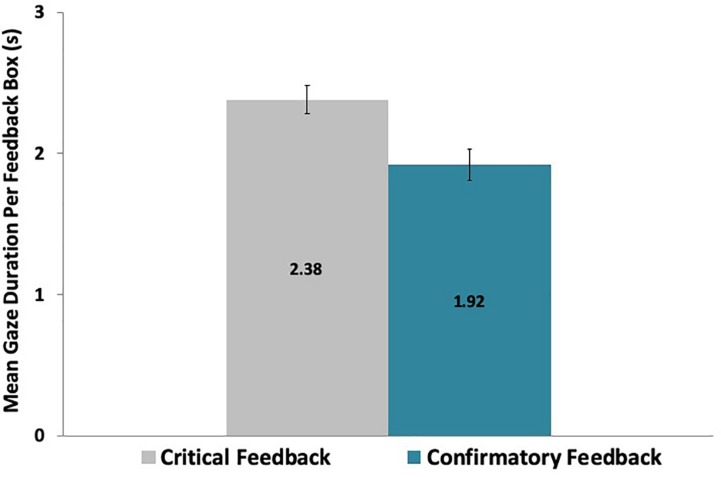
Participants dwelled more on critical than on confirmatory feedback messages across the game.

Analyses of outcome differences were conducted to compare the time participants took to attend to the critical feedback and to the confirmatory feedback across the game. Results of paired-samples *t*-tests revealed that the average gaze duration was significantly longer [*t*(29) = 5.08, *p* < 0.001] for critical (M = 2.38 s, SD = 0.54 s) than for confirmatory (M = 1.92 s, SD = 0.60 s) feedback across the game, as shown in Figure 3. In all figures, error bars represent ± one standard error.

**FIGURE 4:**
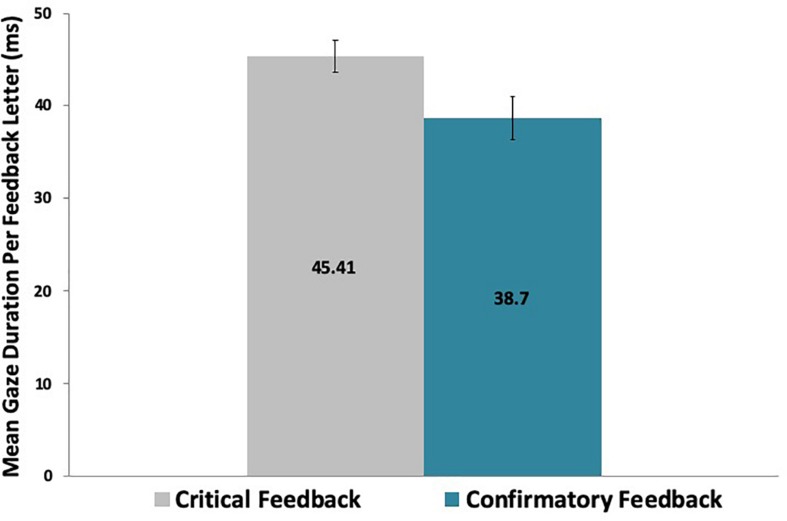
Participants spent significantly more time dwelling on critical than on confirmatory feedback letters across the game.

**FIGURE 5:**
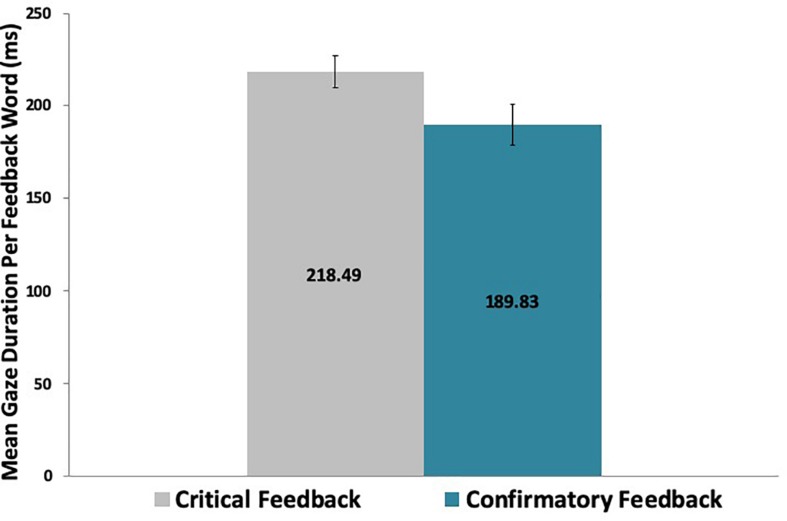
Participants spent significantly more time dwelling on critical than on confirmatory feedback words across the game.

Analyses that took into consideration the length of the feedback message revealed that participants also dwelled longer on critical feedback per letter [*t*(29) = 3.64, *p* < 0.01] and per word [*t*(29) = 3.18, *p* < 0.01] than on confirmatory feedback, as shown in Table 2 and Figures 4, 5.

**TABLE 2:** Means and standard deviations (in seconds) of gaze duration by letter and by word for each feedback valence.

**Measures (*n* = 30)**	**Mean (SD) Gaze**	**Mean (SD) Gaze**
	**Duration per Letter**	**Duration per Word**
Critical	45.41 (9.51)	218.49 (47.40)
Confirmatory	38.70 (12.58)	189.83 (61.54)

The mean values per letter and per word by feedback valence shown in Table 2 are in concordance with prior research regarding the average fixation duration of 200–250 milliseconds (reading across 7–9 letters) for silent reading (Rayner, 1998).

### Do Results Persist When Feedback Revisits Are Discounted?

We also conducted the analyses above without including the fixation durations of the regressions (i.e., the revisits on feedback) to explore whether the difference in dwelling on critical and confirmatory feedback is due to participants returning more often to critical feedback or to participants taking more time to attend to critical feedback the first time around (i.e., before returning to the feedback). Results revealed no significant differences in dwell time [*t*(29) = 0.95, *p* = 0.35] between critical feedback (M = 1.69 s, SD = 0.39 s) and confirmatory feedback (M = 1.59 s, SD = 0.57 s).

Pearson correlations shown in Table 3 were also conducted to explore the associations among these variables. Results suggest that if participants are assigned critical feedback more often, they dwell on it longer per letter and per word. No associations between critical feedback and dwell time per letter and per word on confirmatory feedback were found.

**TABLE 3:** Bivariate correlations between the assigned critical feedback and the mean gaze durations per feedback valence by feedback letter and word.

**Measures**	**Critical Feedback**
Mean Gaze Duration Per Critical Feedback Letter	0.43^∗^
Mean Gaze Duration Per Critical Feedback Word	0.41^∗^
Mean Gaze Duration Per Confirmatory Feedback Letter	0.31
Mean Gaze Duration Per Confirmatory Feedback Word	0.32

*^∗^*p* < 0.05.*

### Is the Mean Number of Gaze Fixations Different Between Valences of Assigned Feedback?

**FIGURE 6:**
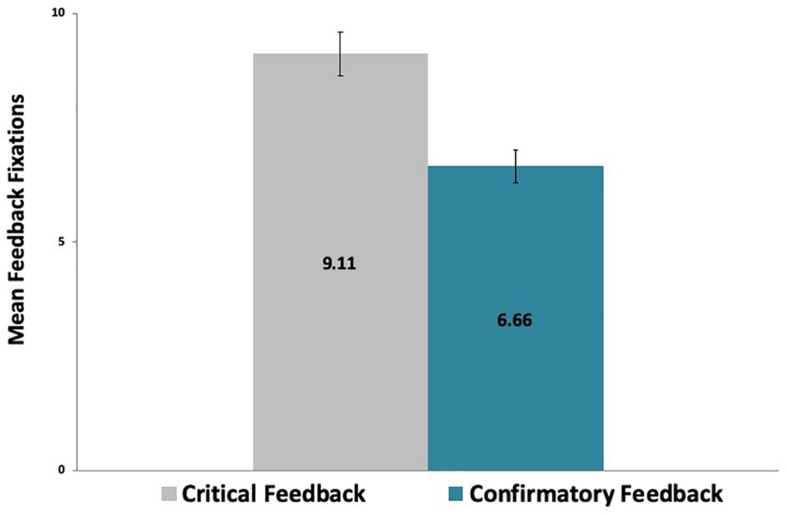
Participants’ fixations on critical feedback messages were significantly more numerous than on confirmatory feedback messages across the game.

A paired-samples *t*-test analysis revealed that the mean number of fixations on critical feedback messages (M = 9.11, SD = 2.57) was significantly larger [*t*(29) = 7.23, *p* < 0.001] than the mean number of fixations on confirmatory feedback messages (M = 6.66, SD = 1.98), as shown in Figure 6. As fixations represent the amount of time that participants focus on the stimulus (i.e., feedback message), this finding points to a decidedly closer attention paid to critical than to confirmatory feedback.

Moreover, there were more gaze fixations on critical feedback (M = 0.17, SD = 0.05) per letter [*t*(29) = 5.75, *p* < 0.001] than on confirmatory feedback per letter (M = 0.13, SD = 0.04). Similarly, there were more gaze fixations on critical feedback (M = 0.82, SD = 0.23) per word [*t*(29) = 5.13, *p* < 0.001] than on confirmatory feedback per word (M = 0.66, SD = 0.20).

### Is the Mean Number of Feedback Revisits Different Between Feedback Valences of Assigned Feedback?

**FIGURE 7:**
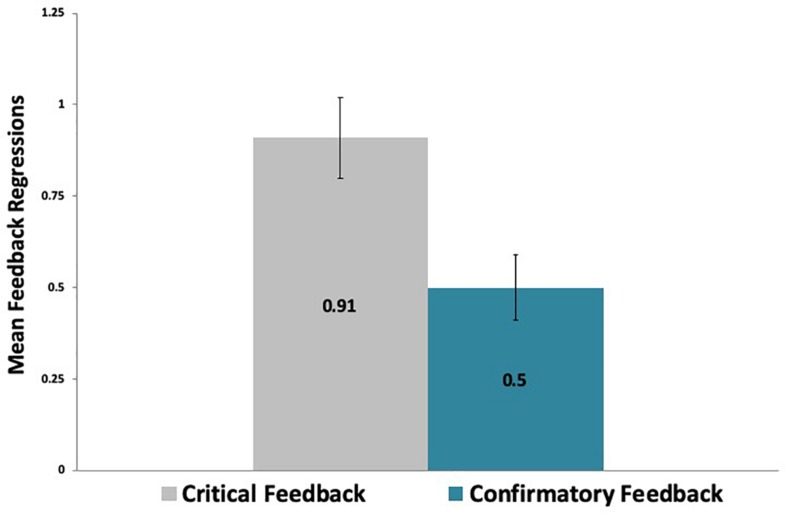
Participants revisited critical feedback messages significantly more often than confirmatory messages.

A paired-samples *t*-test analysis revealed that, on average, participants regressed more [*t*(29) = 3.34, *p* < 0.01] on critical feedback messages (M = 0.91, SD = 0.61) than on confirmatory feedback messages (M = 0.50, SD = 0.51), as shown in Figure 7. As regressions represent the number of times that participants returned to inspect feedback, this finding suggests that participants revisited critical feedback more frequently than confirmatory feedback.

Moreover, participants also tended to revisit more often [*t*(29) = 3.06, *p* < 0.01] the critical feedback per letter (M = 0.02, SD = 0.01) than the confirmatory feedback per letter (M = 0.01, SD = 0.01). They also revisited critical feedback (M = 0.08, SD = 0.06) more often per word [*t*(29) = 2.99, *p* < 0.01] than confirmatory feedback (M = 0.05, SD = 0.05).

## Discussion, Significance, and Limitations

### Do Participants Dwell More on Assigned Critical Feedback Than on Confirmatory Feedback? Do Results Persist When Feedback Revisits Are Discounted?

First, this study showed that undergraduate students spent significantly more time looking at the feedback messages overall as well as at the feedback messages per letter and per word when they attended to critical rather than to confirmatory feedback. However, when the dwell time contribution of the regressions (i.e., revisits on feedback) was discounted, there were no differences between valences in feedback dwell time. This suggests that, when students read the feedback for the first time, before moving their gazes outside the feedback box, they spend the same amount of time reading feedback of both valences. The crucial difference is that students who are assigned their feedback decide to revisit critical feedback significantly more often than confirmatory feedback. In future studies, these results will be compared to the situation in which students can choose their feedback valence.

Taken together, results suggest a deeper processing of critical than of confirmatory feedback, regardless of the feedback valence assigned to the students. This result is in concordance with prior research showing that fixation durations increase with higher mental processing (Velichkovsky et al., 2002). In an eye-tracking study based on a categorization task, participants attended more closely to the stimulus presented during feedback on incorrect trials than on correct trials, as they did not need as much time to process information that they already knew (Watson and Blair, 2008). However, their study focused on dwell time on stimuli, as the stimulus was presented again when feedback was displayed, and it also employed visual (not textual) feedback. The current findings are also supported by the PES adjustment process (Botvinick et al., 2001) predicting a longer dwell time after an error (i.e., critical feedback) than after a correct trial (i.e., confirmatory feedback). Critical feedback could have also come as a surprise to participants, especially as they did not have the option of choosing their feedback valence, leading them to spend more time examining this type of feedback. This possibility is supported by the attentional-orienting theory, which also leads to PES (Houtman and Notebaert, 2013). Furthermore, results could also be explained by the hypercorrection effect, as surprising feedback tends to elicit more attention (Butterfield and Metcalfe, 2006).

Spending more time on critical feedback can be due to the perceived usefulness of critical feedback rather than of confirmatory feedback, especially when participants learn a new skill (e.g., designing posters). This behavior could be related to other factors, such as the content of the feedback (i.e., informative or uninformative) for the same feedback valence. As participants received critical feedback more often than confirmatory feedback in the game, it is possible that they encountered critical uninformative feedback (e.g., “I don’t like fairs”) that could have puzzled them and challenged them to think of what could have been the issue with their poster. Prior research provides some support for this alternative explanation, as it showed that critical uninformative rather than informative feedback was positively associated with participants’ choice to revise their posters (Cutumisu, 2017). This result also echoes other similar finding regarding the dwell time on critical versus confirmatory feedback when feedback is chosen rather than assigned (Cutumisu et al., 2015). Other researchers suggested that participants go through a process of testing several hypotheses during learning, modifying their hypotheses only after encountering an error (Halff, 1975), which may provide support for a prolonged processing of critical feedback in comparison to confirmatory feedback.

Finally, attending more to critical feedback than to confirmatory feedback could also be due to individual differences. Future studies will measure additional factors, such as interpersonal functioning (Formica et al., 2017; Lo Coco et al., 2018). In the context of social feedback, individuals perform more self-focused attention adjustments to deal with criticism than with confirmatory information (Buscher et al., 2012; Vanderhasselt et al., 2015). Research studies have also found that mood can lead individuals to be more open to negative feedback. For instance, a positive mood, compared to a neutral mood, can be used as a tool to minimize ego threat by increasing individuals’ willingness to examine negative feedback. Moreover, the anticipation of receiving negative feedback leads to individuals spending more time reviewing past successes as a mood-boosting way to cope with the stigma of negative feedback (Trope and Neter, 1994; Williams and Ehrlinger, 2017). Recently, an eye-tracking research study found that instruction with negative emotional text design led participants to a deteriorated emotional state after the learning activity (Stark et al., 2018). Additionally, another eye-tracking study found that positive emotional states were associated with better learning outcomes and longer fixation durations on text (Park et al., 2015).

The sample size constitutes one of the limitations of this study. However, this is due to the unique methodology employed in this study, as the experiment could not be designed using the DataViewer to analyze the data automatically. Instead, a video was used in conjunction with the eye tracker, so the Screen Recorder software was employed to record participants’ gazes onto the feedback messages presented in the game. As such, the areas of interest could not be pre-determined, as the game was unique to each participant, depending on each participant’s choices. This led to a laborious manual coding process that made a larger sample prohibitive. However, these results are supported by several Posterlet behavioral studies showing that the amount of time students spend reading feedback correlates with the amount of critical feedback they encounter in the game, meaning that the feedback dwell time inversely correlates with the amount of confirmatory feedback they encounter in Posterlet, as critical and confirmatory feedback measures are complementary.

Another limitation is the lack of an experimental condition in which participants could choose the valence of their feedback. In future research, similar analyses will be conducted to examine data collected from participants who had the opportunity to choose their feedback valence from the animal characters in Posterlet. Would critical feedback be perceived differently than in the current assign condition? Would the deleterious effects of critical feedback (Kluger and DeNisi, 1998; Hattie and Timperley, 2007) be offset by the feedback valence choice that is given to the players in the *Choose* condition? Would the same gaze behavior persist in that case? Also, would some students be inclined to examine critical feedback more closely than other students?

### Is the Mean Number of Gaze Fixations Different Between Valences of Assigned Feedback?

Second, the study investigated whether there were differences in the average fixations on assigned feedback between valences. Results suggest that participants focused more deeply on critical than on confirmatory feedback overall as well as at the level of feedback letters and words. This implies that students tend to pay more attention to critical than to confirmatory feedback when feedback is assigned to them. As fixations indicate situations in which participants are taking in or encoding information (Poole and Ball, 2006), then allocating continued attention to the critical feedback area of the screen would indicate a deeper, sustained processing of the feedback message being read. An agglomeration of fixations on an area of interest (e.g., critical feedback) indicates a prolonged focus of attention on that area.

One possible explanation for this result may be due to the nature of feedback delivery. As students are assigned feedback that is either critical or confirmatory and that they have not requested, they may react differently to this feedback. Future studies will explore this relation in more depth and they will also compare it to the situation in which participants can choose their feedback. Another explanation of these results could be attributed to individuals’ time perspectives (Mannino and Caronia, 2017; Mannino et al., 2017). Specifically, when deciding how to engage with each feedback valence, some individuals may search their memory for similar situations to guide their future behavior (i.e., endorsing a past orientation), while others may consider the future costs and benefits of engaging with feedback (i.e., endorsing a future orientation; Mannino and Caronia, 2017).

One limitation of this measure is that the number of fixations was not differentiated between visits and revisits on feedback messages, as well as between informative and uninformative feedback messages. The current analyses will be repeated to examine whether the different information found in the feedback messages (e.g., informative versus uninformative feedback) influences the results, as well as to separate the contribution of the regressions (i.e., revisits on feedback) when we count the number of fixations on feedback for each valence.

### Is the Mean Number of Feedback Revisits Different Between Valences of Assigned Feedback?

Lastly, results revealed that participants returned significantly more often to attend to critical than to confirmatory feedback messages. This result held at the level of feedback letters and words. The results of the first research question could be interpreted from the lens of the current result: participants not only spent more time per letter and per word of critical feedback compared to confirmatory feedback, but they returned to examine critical feedback more often than confirmatory feedback. This finding aligns with the rest of the results of this study, as it suggests that participants attended to critical feedback more frequently and for a longer time than to confirmatory feedback. It also resonates with prior research suggesting that, during incorrect trials, individuals shift their focus more to error-reduction and hypothesis testing (Watson and Blair, 2008). This may lead to revisiting the critical feedback messages in search of a strategy to apply when designing the next poster.

Perhaps participants are trying to make sense of feedback that they did not request on the poster design task or they are puzzled by the feedback message and thus they are paying repeated attention to it after examining the other pieces of feedback on each of the three posters. This result also suggests that participants may value critical over confirmatory feedback when working on a task, as they learn more from information carried in critical feedback that they did not know already. This could also indicate a greater discernment for critical over confirmatory feedback in solving a new and creative task as digital poster design.

Future research will examine the rate of poster design revisions in relation with critical feedback that is either assigned or chosen by individuals. More fine-grained analyses will separate the fixation frequency between the participants’ first visit on each feedback message and subsequent visits. Future studies will also explore whether attentional engagement with the two feedback valences varies in other more authentic settings, including a social environment where feedback is delivered by peers or instructors. Different populations will be sampled to probe the generalizability of these findings. For instance, it was recently found that children displayed longer latencies and shorter fixation durations than adults (Chen and Tsai, 2015).

## Conclusion and Educational Implications

This study was designed to determine empirically whether participants who were assigned their feedback valence attended more closely to critical feedback than to confirmatory feedback in a digital assessment game. Indeed, results of the different analyses suggest that students attended to critical feedback more often and for a longer period of time than to confirmatory feedback, confirming the initial study hypothesis. Moreover, they dwelled longer on critical rather than on confirmatory feedback per letter and per word, but this difference was driven by the more frequent revisits on critical feedback rather than on confirmatory feedback. These results contribute to increasing our understanding of visual information processing and provide an insight into how individuals process critical and confirmatory feedback, respectively. The study makes the methodological contribution of employing eye tracking to complement behavioral methods that were not sufficient in determining the precise time participants spent looking at each feedback valence. Implications of this research include the development of interactive technologies designed to provide effective feedback. Specifically, this research may inform the design of feedback systems, as well as the delivery of critical and confirmatory feedback (i.e., chosen or assigned), so that students can focus on the type of feedback that helps them improve their outcomes most effectively. Lastly, this study brings more evidence to support the use of eye-tracking online methods for learning processes over behavioral methods alone to investigate feedback processing and to understand the factors underlying a deeper processing of critical feedback.

## Data Availability

The datasets generated for this study are available by request to the corresponding author.

## Ethics Statement

Ethics Application: Eye tracking students’ gameplay in Posterlet, Pro00059774, Research Ethics Board 2, University of Alberta Research Ethics Office.

## Author Contributions

MC designed and implemented the study, created the ethics application, collected and assembled the data, coordinated and contributed to the coding of the eye-tracking data, analyzed the data, interpreted the results, and wrote the manuscript. K-LT contributed to the data collection, eye-tracker setup, coordination and coding of the eye-tracking data, and manuscript editing. TS, SC, LE, RM, VK, K-LT, and MC contributed to coding of the eye-tracking data. MC, K-LT, TS, SC, LE, RM, and VK critically revised the manuscript for important intellectual content and approved the final manuscript.

## Conflict of Interest Statement

The authors declare that the research was conducted in the absence of any commercial or financial relationships that could be construed as a potential conflict of interest.
